# Valor Prognóstico da Pontuação PRECİSE DAPT em Pacientes MINOCA com Síndrome Coronariana Aguda

**DOI:** 10.36660/abc.20240307

**Published:** 2024-06-21

**Affiliations:** 1 Universidade Federal de São Paulo São Paulo SP Brasil Universidade Federal de São Paulo (UNIFESP), São Paulo, SP – Brasil

**Keywords:** Síndrome Coronariana Aguda, MINOCA, Infarto do Miocárdio

Nas últimas décadas, o manejo terapêutico do infarto agudo do miocárdio (IAM) progrediu substancialmente devido às crescentes inovações. Desta forma, o desenvolvimento e a compreensão de biomarcadores, como por exemplo, a troponina, apresentou-se como um marcador diagnóstico mais preciso da lesão miocárdica e é agora a pedra angular das definições contemporâneas de IAM. Esses achados facilitaram o desenvolvimento de terapias de reperfusão para o infarto do miocárdio com supradesnivelamento do segmento ST (IAMCSST), inicialmente envolvendo terapia trombolítica intravenosa e, subsequentemente, intervenções coronárias percutâneas primárias, quando as primeiras demonstraram ser menos eficazes na restauração do fluxo sanguíneo coronário angiográfico e na melhoria dos resultados clínicos.^1^ Entretanto, cerca de 5% a 6% dos casos de IAM apresentam-se com coronárias sem lesões obstrutivas (ou seja, lesões com mais de 50% de estenose), doravante denominado MINOCA, do inglês, *myocardial infarction with nonobstructive coronary arteries* (infarto do miocárdio com artérias coronárias não obstrutivas). Dados de grandes registros sugerem prevalência de MINOCA entre 2 e 10%, dependendo da coorte estudada e dos critérios diagnósticos utilizados. O maior desses estudos examinou pacientes com IAMSSST do registro CRUSADE e relatou que o sexo feminino e em idade mais jovem, foram os únicos preditores clínicos independentes de MINOCA.^2 – 4^

Um aspecto importante na avaliação de pacientes com MINOCA aparente é excluir causas não isquêmicas, como embolia pulmonar, insuficiência renal crônica, insuficiência cardíaca crônica, miocardite, cardiomiopatias (infiltrativa, takotsubo, periparto…), acidente vascular cerebral, choque séptico, síndrome do desconforto respiratório agudo, trauma cardíaco (incluindo iatrogênico), queimaduras graves, agentes quimioterápicos e exercícios extenuantes.^5^ O espasmo da artéria coronária é outra causa importante de oclusão transitória de uma artéria epicárdica e a marca registrada da angina variante ou vasospástica. A presença de alterações transitórias do segmento ST durante a dor torácica que responde à terapia com nitrato é consistente com o diagnóstico. Claramente, mais estudos são necessários nesta área, especialmente porque a angina vasospástica está associada a risco aumentado de IAM/morte.^6 – 10^

Por sua vez, o escore PRECISE-DAPT de cinco itens, integrando idade, hemoglobina, contagem de leucócitos, depuração de creatinina e sangramento prévio, prediz o risco de sangramento em pacientes em terapia antiplaquetária dupla (DAPT) após o implante do stent. Nesta análise, prospectou-se avaliar o risco de sangramento entre pacientes que recebem monoterapia com ticagrelor a partir de 1 mês após implante de stent coronário. A capacidade do escore de prever sangramento pelo critério BARC ( *Bleeding Academic Research Consortium* ) 3 ou 5 foi avaliada e comparada entre pacientes em monoterapia com ticagrelor (estratégia experimental) ou DAPT padrão (estratégia de referência) a partir de 1 mês após o implante do stent farmacológico.^11^ A análise da curva de decisão mostrou benefício líquido usando o PRECISE-DAPT para orientar a avaliação do risco de sangramento em ambas as estratégias de tratamento.^12^

Em interessante publicação, motivo deste editorial, os autores avaliaram em estudo retrospectivo e observacional, 7.300 pacientes internados com diagnóstico de IMCSST ou sem supradesnivelamento do segmento ST (IAMSSST).^13^ Um subconjunto de 741 indivíduos recebeu diagnóstico de síndrome coronariana aguda e MINOCA. A angiografia coronária foi realizada em todos os pacientes. Pacientes que não apresentavam estenose coronariana de 50% ou mais em qualquer artéria coronária na angiografia coronária e que não foram diagnosticados com dissecção espontânea da artéria coronária, miocardite ou cardiomiopatia de takotsubo foram classificados como MINOCA. O escore PRECISE-DAPT, idade, depuração de creatinina, contagem de leucócitos, hemoglobina e histórico de sangramento prévio foram calculados para cada caso. Neste estudo, foi apontado que um escore PRECISE-DAPT alto é um preditor independente de eventos graves para o período intra-hospitalar e de longo prazo em pacientes MINOCA com síndrome coronariana aguda.

Desta forma, destaca-se que a pontuação PRECISE-DAPT pode ser calculada de forma rápida e fácil, fornecendo uma classificação de risco adequada. Adicionalmente, os autores relatam algumas limitações, fundamentalmente por ser um estudo retrospectivo e observacional. Portanto, embora com interessantes perspectivas de incorporação para a prática clínica, estes resultados e a duração dos eventos devem ser interpretados com cautela.
