# Supplementary material for: Valor Prognóstico da Pontuação PRECİSE DAPT em Pacientes MINOCA com Síndrome Coronariana Aguda
Source: Arq Bras Cardiol. 2024 Jun 21;121(5):e20240307. [Article in Portuguese] doi: 10.36660/abc.20240307 (PMC11216326; doi:10.36660/abc.20240307)
Supplement: Translation

# Translation: Prognostic Value of PRECİSE DAPT Score in MINOCA Patients with Acute Coronary Syndrome

**DOI:** 10.36660/abc.20240307i

**Affiliations:** São Paulo SP Brazil Universidade Federal de São Paulo (UNIFESP), São Paulo, SP – Brazil

**Keywords:** Acute Coronary Syndrome, MINOCA, Myocardial Infarction

In recent decades, the therapeutic management of acute myocardial infarction (AMI) has progressed substantially due to increasing innovations. Thus, the development and understanding of biomarkers, such as troponin, has emerged as a more accurate diagnostic marker of myocardial injury and is now the cornerstone of contemporary definitions of AMI. development of reperfusion therapies for ST-segment elevation myocardial infarction (STEMI), initially involving intravenous thrombolytic therapy and later primary percutaneous coronary interventions when the former found to have less effect on restoring coronary blood flow. angiography and improving clinical results.^1^However, approximately 5% to 6% of AMI cases present with coronary arteries without obstructive lesions (i.e., lesions with more than 50% stenosis), hereinafter referred to as MINOCA, myocardial infarction with nonobstructive coronary arteries. Data from large registries suggest a prevalence of MINOCA between 2 and 10%, depending on the cohort studied and the diagnostic criteria used. The largest of these studies examined patients with STEMI from the CRUSADE registry and reported that female sex and younger age were the only independent clinical predictors of MINOCA.^2 – 4^

An important aspect in the evaluation of patients with apparent MINOCA is to exclude non-ischemic causes, such as pulmonary embolism, chronic renal failure, chronic heart failure, myocarditis, cardiomyopathies (infiltrative, takotsubo, peripartum…), stroke, septic shock, syndrome from acute respiratory distress, cardiac trauma (including iatrogenic), severe burns, chemotherapy agents, and strenuous exercise.^5^Coronary artery spasm is another important cause of transient occlusion of an epicardial artery and the hallmark of variant or vasospastic angina. The presence of transient ST-segment changes during chest pain that responds to nitrate therapy is consistent with the diagnosis. Clearly, more studies are needed in this area, especially because vasospastic angina is associated with an increased risk of AMI/death.^6 – 10^

In turn, the five-item PRECISE-DAPT score, integrating age, hemoglobin, leukocyte count, creatinine clearance, and previous bleeding, predicts the risk of bleeding in patients on dual antiplatelet therapy (DAPT) after stent implantation. In this analysis, we aimed to evaluate the risk of bleeding among patients receiving monotherapy with ticagrelor from 1 month after coronary stent implantation. The ability of the score to predict bleeding according to the BARC (Bleeding Academic Research Consortium) criteria 3 or 5 was evaluated and compared between patients on monotherapy with ticagrelor (experimental strategy) or standard DAPT (reference strategy) from 1 month after implantation of the drug-eluting stent.^11^Decision curve analysis showed net benefit using PRECISE-DAPT to guide bleeding risk assessment in both treatment strategies.^12^

In an interesting publication, the reason for this editorial, the authors evaluated, in a retrospective and observational study, 7,300 patients hospitalized with a diagnosis of STEMI or without ST-segment elevation (STEMI).^13^A subset of 741 individuals received a diagnosis of acute coronary syndrome and MINOCA. Coronary angiography was performed in all patients. Patients who did not have coronary stenosis of 50% or more in any coronary artery on coronary angiography and who were not diagnosed with spontaneous coronary artery dissection, myocarditis, or takotsubo cardiomyopathy were classified as MINOCA. The PRECISE-DAPT score, age, creatinine clearance, white blood cell count, hemoglobin, and previous bleeding history were calculated for each case. In this study, it was pointed out that a high PRECISE-DAPT score is an independent predictor of serious events for the in-hospital and long-term periods in MINOCA patients with acute coronary syndrome.

In this way, it is highlighted that the PRECISE-DAPT score can be calculated quickly and easily, providing an adequate risk classification. Additionally, the authors report some limitations, mainly because it is a retrospective and observational study. Therefore, although with interesting prospects for incorporation into clinical practice, these results and the duration of events must be interpreted with caution.
